# Case report: Invasive fungal infection in a patient with a rare CVID-causing gene (*TNFRSF13B*) mutation undergoing AML treatment

**DOI:** 10.3389/fonc.2023.1017230

**Published:** 2023-03-15

**Authors:** Carine Tabak, Stephen Hyter, Abdulraheem Yacoub, Kenneth Byrd, Joseph McGuirk, Andrew K. Godwin, Haitham Abdelhakim

**Affiliations:** Division of Hematologic Malignancies and Cellular Therapeutics, University of Kansas School of Medicine, Kansas City, MO, United States

**Keywords:** acute myeloid leukemia, invasive fungal infections, *TNFRSF13B*, CVID, NGS

## Abstract

Acute myeloid leukemia (AML) is a complex diagnosis that puts patients at a higher risk for developing infections, particularly invasive fungal infections (IFI). Mutations in *TNFRSF13B* have been shown to cause dysfunction in B-cell homeostasis and differentiation, making it a risk factor for developing immunodeficiency syndromes. In this case, a male patient in his 40s presented to our emergency department (ED) with symptoms leading to a diagnosis of AML with concurrent mucormycosis of the lungs and sinuses. Targeted next generation sequencing (NGS) of the patient’s bone marrow showed, among other variants, a loss of function mutation in the *TNFRSF13B* gene. While most patients present with fungal infections after prolonged periods of neutropenia associated with AML treatment, this case presented with IFI at diagnosis without neutropenia suggesting an immunodeficiency syndrome. The concurrent IFI and AML diagnoses create a delicate balance between treatment of the infection and the malignancy. This case highlights the risk of infection in patients receiving chemotherapy, especially those with unrecognized immunodeficiency syndromes, and emphasizes the importance of NGS for prognosis and treatment.

## Background

Acute myeloid leukemia (AML) is a hematological malignancy that is characterized by abnormal proliferation and infiltration of the blood and bone marrow by cells of the hematopoietic lineage (1). Over the past few decades, advanced therapies and a better understanding of the pathology of AML have turned the fatal disease into a treatable condition. However, many comorbidities can still make AML management aggressive and complex. Due to the hematological nature of the cancer, AML patients often present with a weakened immune system, making them more likely to develop opportunistic infections.

An AML diagnosis puts patients at a high risk for developing infections in general and invasive fungal infections (IFI) in particular. IFI is a major cause for mortality in patients with leukemia (2). The incidence of IFI in patients with AML has been reported to be as high as 12% (3). Due to the limited efficacy of most antifungal agents against IFIs, a risk-adapted strategy with an emphasis on prophylactic treatment is often implemented with an AML diagnosis (4). Candidemia and aspergillosis pathogens are the most common cause of fungal infections. However, the spectrum of fungal infections has shifted dramatically to include more fluconazole-resistant non-*Aspergillus* and non-*Candida albicans* species over the last few decades (5). The infection can become disseminated and can affect various organ systems depending on the pathogen, most prevalent are infections of the lungs, sinuses and of the bloodstream (2). Patients have a probability of 11.1% of developing IFI infections within 100 days of an AML diagnosis and experience a 35% cause-specific mortality due to the infection (6, 7). Furthermore, the diagnosis leads to substantial increases in health expenditure and hospital stay length (8). The regular use of anti-fungal prophylaxis has improved the outcomes of patients with AML (9, 10). However, breakthrough infections are not uncommon (11, 12).

Various genetic mutations could put patients, including patients with AML, at a higher risk for developing opportunistic infections. Mutations in *TNFRSF13B* have been noted as a possible risk factor to developing immunodeficiency syndromes, specifically CVID-like (common variable immune deficiency) manifestations (13). CVID is characterized by an inability to mount a full antibody immune response, and is associated with recurrent bacterial infections, autoimmunity, and lymphoproliferative disorders (14).

*TNFSF13B* encodes for B cell-activating factor (BAFF), which binds three TNF receptors expressed on B cells known as BAFFR (BAFF receptor, encoded by *TNFRS13C*), BCMA (B cell maturation protein A, encoded by *TNFRSF17*), and TACI (transmembrane activator and CAML interactor, encoded by *TNFRSF13B*) (13). Of the three sequence variants, mutations in TACI (encoded by *TNFRSF13B*) are of note due to their association with immunodeficiency syndromes. TACI loss of function mutations have been reported in 7-10% of patients diagnosed with CVID and have also been reported in IgA deficiency (14). TACI is a tumor necrosis factor receptor superfamily member that is expressed on peripheral B-cells, with the highest grade of expression on CD27+ B-cell subset 3. TACI binds two ligands that influence cell proliferation: a proliferation inducing ligand (APRIL) and B-cell activating factor (BAFF) (15). APRIL has been tied to impaired class switching to IgA7 in mice models and T-cell independent class switch recombination in functional studies (13). Therefore, variants in *TNFRSF13B* gene can lead to critical dysfunction in B-cell homeostasis, class-switch recombination, antibody secretion and plasma cell differentiation (16, 17). Due to this association with immune dysfunction, variants in *TNFRSF13B* would potentially place patients with AML at higher risk for developing opportunistic infections, such as invasive fungal infections. This study was IRB approved.

## Clinical course

**Figure 1:**
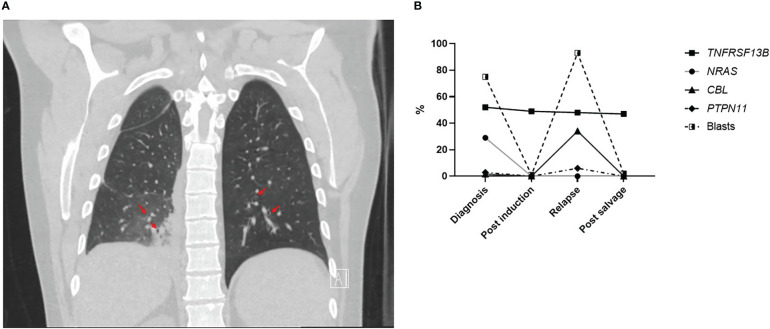
**(A)** Coronal section of lung CT scan showing bronchiectasis (red arrows). **(B)** Percentage of different gene variants detected on NGS and percentage of AML blasts on morphology from bone marrow samples evaluated at different timepoints during the treatment course.

A male patient in his 40s presented to the ED with fever, malaise and mouth lesions after a recent dental procedure. The patient had a past medical history of depression and recurrent upper respiratory tract infections, not requiring hospitalization, and no personal history of malignancy or autoimmune disease. His surgical history includes appendectomy and cholecystectomy. Family history was positive for diabetes in his father and brother, and negative for any history of malignancy. Upon ED laboratory evaluation, the patient was found to have hyperleukocytosis with suspicion for acute leukemia. The patient was found to be hypoxic on admission and needed supplemental oxygen. CT scan of the lungs showed evidence of bilateral infiltration suggestive of fungal pneumonia but could not rule out leukemic infiltration. Lung CT scan also showed bronchial dilatation and wall thickenings (**Figure 1A**). Respiratory viral panel including RSV, COVID and flu testing were negative. Due to hyperleukocytosis and spontaneous tumor lysis syndrome, the patient was started on hydroxyurea for cytoreduction. Treatment with broad coverage with antibiotics and posaconzaole was initiated. He underwent bronchoscopy with bronchoalveolar lavage which was inconclusive with persistent negative cultures.

The bone marrow biopsy confirmed AML diagnosis with core binding factor. FISH studies showed results consistent with acute myeloid leukemia with 46,XY, inv(16)(p13.1.q22). NGS was performed using a 141 myeloid-focused gene panel and showed a pathogenic *NRAS* mutation. These prognostic markers put the patient in the favorable risk group. The patient was started on daunorubicin/cytarabine (standard 7 + 3 regimen) + gemtuzumab after improvement in tumor lysis parameters with hydroxyurea cytoreduction. The patient’s clinical picture deteriorated with persistent fever, sinus pain, development of hypoxic respiratory failure after one week. Repeat CT chest showed progression of nodular multifocal pneumonia suggestive fungal pneumonia. Sinus MRI confirmed acute invasive fungal sinusitis with right orbital spread. The patient was started on liposomal amphotericin, isavuconazole and micafungin. To debulk and identify the infection, the patient underwent debridement with septoplasty, right maxillary antrostomy, right total ethmoidectomy and sphenoidectomy with tissue debridement. Two days later, the patient underwent nasal endoscopy with debridement, endoscopic orbital decompression and debridement. Bronchoscopy was repeated with bronchoalveolar lavage and transbronchial biopsy. Both the sinus and respiratory specimens confirmed angioinvasive fungal infection, histologically most consistent with Mucorales species, but with negative cultures. Triple anti-fungal therapy was continued. Bone marrow biopsy showed persistent AML disease and patient opted not to receive chemotherapy treatment and was discharged home.

After 2 months, the patient had significant clinical improvement, He re-established care with the leukemia clinic. CT showed improvement in multifocal pneumonia, with mild residual opacities possibly from scarring and/or recurrent infection. Further follow-up showed remission of AML and recovery of blood counts. Fungal infection was persistent on nasal endoscopy and biopsy. He opted to delay chemotherapy and continue anti-fungal treatments with close follow up. AML relapsed 8 months from the original diagnosis but is back in remission after reinduction chemotherapy with mitoxantrone, etoposide and cytarabine (MEC). Patient stayed on antifungal therapy, and he tolerated the treatment without a flare of the IFI. He proceeded with allogeneic stem cell transplant with reduced intensity conditioning from a full matched sibling 11 month after the initial diagnosis. Patient achieved 98%, 100% donor chimerism at D60,100 post-transplant respectively. However, he had AML relapse 6 months after transplant.

The presence of invasive fungal infection at AML diagnosis before starting treatment without indication of prior prolonged neutropenia prompted assessment of immunoglobulins levels. The patient had low IgG levels when checked after complete recovery from chemotherapy at multiple visits (610-628 mg/dl). IgA was low normal (41 mg/dl) and IgM was within normal limits at this time (157 mg/dl). Absolute neutrophil, monocyte and lymphocyte counts were within normal level when AML was in remission (3400-5900/μL, 500-1100/μL,900-2200/μL, respectively). Furthermore, NGS results at initial diagnosis showed a likely pathogenic loss-of-function variant in the *TNFRSF13B* gene (c.311G>A/p.C104Y) at a variant allelic fraction (VAF) of 52%. Although germline testing was not performed, this particular lesion involves the same codon as the well-known C104R familial variant linked to CVID, suggesting the patient had unrecognized CVID prior to AML diagnosis (13, 18). Additional evidence for the non-somatic nature of this *TNFRSF13B* variant is the VAF stability seen during remission and relapse stages of this patient’s disease in comparison to other pathogenic variants associated with AML (**Figure 1B**). In addition to hypogammaglobulinemia, patients had mild supraclavicular, mediastinal, abdominal lymphadenopathy with slightly large spleen (10.5 cm) at diagnosis that remained unchanged during AML remission. All lymph nodes were subcentimeteric so further diagnostic intervention was not pursued. Lymphoproliferation with mild splenomegaly and lymphadenopathy is predominant in CVID patients (14). Interestingly, repeat CT scan 6 months post-allogeneic stem cell transplant showed resolution of all lymphadenopathy and splenomegaly. Moreover, IgG level normalized (894 mg/dl) which coincided with the presence of full donor lymphoid chimerism concurrently with drop in donor myeloid chimerism and AML relapse. This highlights that hypogammaglobulinemia and lymphoproliferation are likely unrelated to the AML diagnosis and likely associated with CVID that was cured after hematopoietic stem transplant and replacement of the patient’s *TNFRSF13B* mutated lymphoid system.

## Discussion

Several scientific advancements over the last decade have improved our understanding of the genetic diversity of AML and have led to the development of new therapies (19). The goal of induction chemotherapy treatment in patients with AML is achieving remission and is associated with severe neutropenia and immunosuppression. This puts patients with AML at higher risk for IFI, which usually develops after a prolonged neutropenic period (3). Nevertheless, the patient in this case presented with IFI at diagnosis with no prior prolonged neutropenic period. This presentation raised the question of an immunodeficiency syndrome that would make him susceptible to infections and his risk for infections further surged with development of AML. While we cannot prove that the *TNFRSF13B* mutation caused the infection, the diagnosis of the IFI in the presence of preexisting bronchiectasis, hypogammaglobulinemia and the lack history of prior neutropenia make it highly likely that the mutation played a role at least in the development of this life-threatening infection.

Various immune deficiency syndromes, such as CVID, can further increase the risk of infections in patients receiving chemotherapy. Loss of function mutations in *TNFRSF13B*, specifically, have been linked to immune deficiency and dysfunction due to dysregulation of B-cell homeostasis, differentiation, and antibody secretion (14). Yet, it is uncommon for an individual to present with AML and concurrently be diagnosed with an immunodeficient mutation, as with the presented case.

Immunodeficiency adds an additional challenge to treating invasive fungal infections in patients with an AML diagnosis. Both the nature of the disease and the associated treatments induce an immunocompromised state in patients that leaves them more vulnerable for infections. On the other hand, patients diagnosed with IFI may be forced to delay chemotherapy for their AML to complete treatment for the infection in order to avoid a prolonged period of neutropenia. Without proper induction and consolidation therapy, patients are extremely unlikely to reach complete remission of their malignancy. This creates a delicate balance between the risk and benefit of treating the malignancy and controlling the fungal infection.

This case highlights the high risk of infection in patients with AML receiving intensive chemotherapy, especially in the rare instance of a concurrent immunodeficiency syndrome. Moreover, it highlights the role of NGS as a powerful tool not only for delineating prognosis of patients with AML but also capturing germline mutations with potential implications of infectious complications during treatment.

## Data availability statement

The original contributions presented in the study are included in the article/supplementary material. Further inquiries can be directed to the corresponding author.

## Ethics statement

The studies involving human participants were reviewed and approved by University of Kansas Medical Center. Written informed consent for participation was not required for this study in accordance with the national legislation and the institutional requirements.

## Author contributions

CT and HA designed the study and wrote the manuscript. AG, JM and SH provided data, and interpretation and contributed to writing and reviewing the manuscript. AY and KB provided data and interpretation and reviewed the manuscript. All authors contributed to the article and approved the submitted version. 
